# The effect of COVID-19 on the non-COVID health outcomes of crisis-affected peoples: a systematic review

**DOI:** 10.1186/s13031-024-00592-7

**Published:** 2024-04-25

**Authors:** N. Thompson, K. W. Y. Kyaw, L. Singh, J. C. Cikomola, N. S. Singh, Bayard Roberts

**Affiliations:** 1 Faculty of Public Health and Policy, London School of Hygiene and Tropical Medicine, London, UK; 2 Faculty of Medicine, Université Catholique de Bukavu, Democratic Republic of the Congo, Central African Republic

**Keywords:** COVID-19, Refugees, Forcibly displaced, Humanitarian, Health services

## Abstract

**Background:**

The COVID-19 pandemic posed considerable risks to populations affected by humanitarian crises in low- and middle-income countries (LMICs). However, there is limited understanding of how the pandemic may have affected non-COVID health outcomes among crisis-affected populations. Our aim was to examine the evidence on the impact of the COVID-19 pandemic on non-COVID-19 health outcomes for crisis-affected populations in LMICs.

**Methods:**

A systematic review methodology was applied following PRISMA guidelines. Eligibility criteria were: crisis-affected populations in LMICS; COVID-19; and all health topics, except for sexual and reproductive health which was covered in a linked review. Five bibliographic databases and additional grey literature sources were searched. The search period was from 2019 to 31 July 2022. Eligible papers were extracted and analysed using a narrative synthesis approach based on the study objectives and relevant health access and systems frameworks. A quality appraisal was also conducted.

**Findings:**

4320 articles were screened, and 15 eligible studies were identified and included in this review. Ten studies collected health outcomes data. Eight related to mental health, which generally showed worse mental health outcomes because of the pandemic, and pandemic-related stressors were identified. Two studies assessed physical health outcomes in children, while none addressed physical health outcomes among adults. Nine studies reported on access to healthcare, revealing worse access levels due to the pandemic and noting key barriers to care. Seven studies reported on the impact on health systems, with key challenges including reduced and distorted health care funding, reduced staff capacity, interrupted medicines and supplies, weak information and mixed-messaging, and weak leadership. All fifteen studies on the social determinants of health, particularly highlighting the effect of increasing poverty, the role of gender, and food insecurity on health outcomes. The quality of papers was limited overall.

**Conclusion:**

This review found some limited evidence indicating negative mental health effects, increased barriers to accessing care, damage to health systems and magnified impacts on the social determinants of health for crisis-affected people during the COVID-19 pandemic. However, the small number and limited quality of the studies make the overall strength of evidence quite weak.

**Supplementary Information:**

The online version contains supplementary material available at 10.1186/s13031-024-00592-7.

## Background

Crisis-affected populations include individuals, groups, and communities directly or indirectly affected by a humanitarian crisis such as armed conflicts and natural disasters. They encompass internally displaced persons (IDPs), refugees, asylum-seekers, stateless individuals, entrapped and other non-displaced crisis-affected populations, and those affected by extreme weather events and natural disasters who require humanitarian assistance [1]. In 2019, around 16% of the global population lived in countries experiencing protracted crises, and 215.6 million people were estimated to need humanitarian assistance [2]. 79.5 million people were forcibly displaced by armed conflict and mass disruption, the majority as IDPs and refugees (including asylum seekers), and 85% of whom lived in low- and middle-income countries (LMICs) [2]. By the end of 2022, this figure had risen to 108.4 million people [3]. 

Crisis-affected populations were identified early in the COVID-19 pandemic as disproportionately vulnerable to the direct and indirect effects of the COVID-19 crisis [4, 5]. This is because crisis-affected populations commonly experience worse health outcomes as a result of disease outbreaks due to interrupted access to health care, interrupted food supplies, damaged public health services and health systems, overcrowding, and increasing poverty [1, 4, 6–8]. Armed conflicts and resultant forced migration are also increasingly protracted which extends exposure to these risk factors and prolongs worse health outcomes. The COVID-19 pandemic could have impacted crisis-affected populations directly through COVID-19 infections but, importantly, also indirectly through affecting other health outcomes and impeding access to health care. Evidence from other disease outbreaks, most notably the West Africa Ebola outbreak from 2014 to 2016, highlighted these indirect health effects from epidemics [9–11]. There have been reviews on the mental health effects of COVID-19 on international migrants [12]. There has also been a systematic review we published on the impact of COVID-19 on sexual and reproductive health (SRH) among crisis-affected population in LMICs [13]. However, to the best of our knowledge, there has been no systematic review of the effects of COVID-19 on non-COVID health outcomes (other than SRH) among crisis-affected populations in LMICs. There is also a need to examine how COVID-19 influenced these outcomes, such as through impeding access to health care, affecting health system performance, and magnifying social determinants of health. It is also valuable to focus on LMICs because the vast majority of crisis-affected populations globally live in LMICs and the resources and health system capacities in LMICS to respond to crises are very different to those in high-income countries.

The aim of our systematic review was to examine the evidence on the impact of the COVID-19 pandemic on non-COVID-19 health outcomes for crisis-affected populations in LMICs. The specific objectives were to examine: (i) the impact of the COVID-19 pandemic on non-COVID-19 health outcomes among crisis-affected populations; (ii) the effect of the COVID-19 pandemic on access to healthcare by crisis-affected populations; (iii) the effect of the COVID-19 pandemic on health systems in crisis-affected settings; and (iv) how the COVID-19 pandemic influenced social determinants of health in crisis-affected populations.

## Methods

We used a systematic review design and followed the Preferred Reporting Items for Systematic Reviews and Meta-Analyses (PRISMA) guidelines (Supplementary Material 1).

### Eligibility criteria

The eligibility criteria in this study are summarised in Table 1. We excluded studies on SRH as these were covered in our related review [13]. 

**Table 1:** Inclusion and exclusion criteria

Category	Included	Excluded
Population/context of interest	Crisis-affected populations: IDPs, refugees, asylum seekers, stateless individuals, entrapped and other non-displaced crisis-affected populations, and those affected by extreme weather events and natural disasters as per UN definitions. Crisis-affected populations based in LMICs, as per World Bank country classification [14].	Studies where COVID-19 is not an exposure. Studies of host populations, with no reference to crisis. Studies of ‘Migrants’ with no reference to populations forcibly displaced by humanitarian crises.
Outcomes of interest	All health condition/disease outcomes Health systems and services Social determinants of health	Sexual and Reproductive Health studies (as this was addressed in our separate study [13]). Studies of COVID-19 health outcomes without reference to systemic effects, or other disease outcomes
Study design & publication type	All primary research studies assessing one or more of: health outcomes, health access, health systems and social determinants of health. All quantitative, qualitative, and mixed-method study designs. Published in academic peer review journals or grey literature.	Editorials, letters, commentaries, books, book chapters, systematic reviews, conference proceedings, literature reviews, opinion pieces, news articles, letters to the editor
Language	English language only	Non-English language publications
Date	From 2019 to 31 July 2022	Prior to 2019

### Search strategy, selection, and extraction

We conducted searches of the published academic and grey literature. Just two categories of search terms were used to ensure sensitivity. The first relates to crisis-affected populations and the second to COVID-19. Studies were then excluded at the screening stage if set in high-income countries (according to World Bank criteria [14]). The full search terms are given in Supplementary Material 2. The bibliographic databases searched for the published literature were Medline, Embase, Global Health, PsychInfo, and IBSS. The grey literature sources included: Google, OpenGrey, Médecins Sans Frontières Science Portal, International Rescue Committee Research, ReliefWeb, ALNAP, International Committee of the Red Cross, and United Nations High Commissioner for Refugees. Eligible published and grey literature studies were collated into Endnote citation software and screened for duplicates. After duplicate removal, the remaining titles and abstracts were double screened for eligibility. We then imported the included studies into Covidence systematic review software for full-text review, data extraction and analysis. The extraction variables included standard ones (e.g. author/date, setting/population, aim, sample, design/methods, outcomes) and those specific to this review on access to care, health systems, and social determinants (please see Tables 4, 5 and 6 for the types of variables used for extraction). The end date for the search was 31 July 2022.

### Analysis and quality appraisal

We conducted a narrative synthesis analysis according to the specific objectives [15]. Access to care was assessed using Penchansky and Thomas’s five parameters: Affordability, Availability, Accommodation, Acceptability, and Accessibility [16]. A sixth parameter, ‘Entitlement, was added to capture any refugee-specific legal barriers relating to healthcare service utilisation as conceptualised by Yang et al. [17] We chose the Penchansky and Thomas framework because it captures in a simplified form the characteristics and expectations of both the health care providers and users. In addition, it has been widely and successfully used. The impact on health systems was categorised across the six pillars of health systems as defined by the WHO [18]. For the social determinants, we broadly followed those used by Dahlgren and Whitehead [19], presenting the determinants most commonly arising in the eligible studies. For quality appraisal of the eligible studies, the observational and cohort studies were appraised using the Newcastle Ottawa Quality Assessment tool [20], and the qualitative studies were appraised using the CASP qualitative research checklist [21]. 

## Results

### Study selection and characteristics

**Fig. 1:**
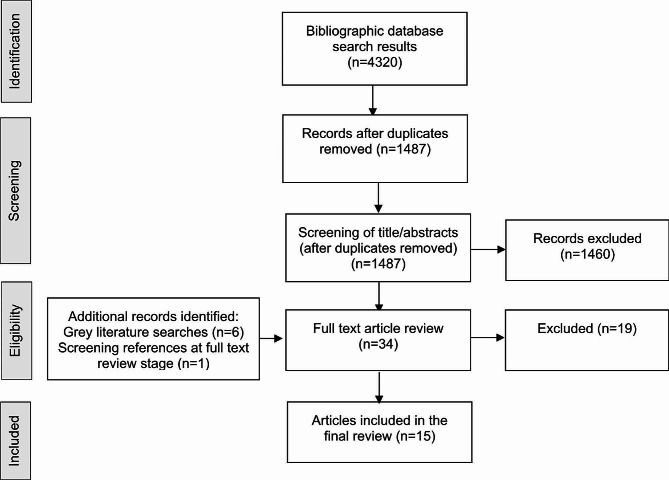
Prisma flow diagram for study screening

The results of the search and screening process are shown in Fig. 1. Our search of the bibliographic databases returned 4320 studies. After removing duplicates, 1487 remained. After screening of titles/abstracts, 1460 were removed. Thirty-four titles were assessed for eligibility for full-text review and nineteen studies were excluded for one of four reasons: wrong population, wrong outcome assessed, not primary research, and wrong setting. Fifteen studies were included in the final review and study characteristics are shown in Table 2 [22–36]. All the studies were from the published literature. Seven were purely quantitative studies (4 cross-sectional surveys, 3 cohort studies), 5 were purely qualitative studies, and 3 were mixed methods studies (cross-sectional surveys and qualitative research). Twelve studies were with refugees, with one of these studies also including host populations and another of these studies also including NGO staff. There were two studies with IDPs. One study was with humanitarian workers.

**Table 2:** Summary of study characteristics (*N* = 15)

Author, year [ref]	Study design	Study population and setting	Study sample	Study aim	Collected	Health Outcome	Access to Care	Health System	Social Determ.
Akhtar 2021 [22]	Observational cohort (adapted from RCT)	Syrian refugees living in Azraq refugee camp, Jordan	410 randomly sampled and screened refugees	To determine the impact of the COVID-19 pandemic on the mental health of Rohingya refugees in Bangladesh	July 2019 – November 2020	Yes	No	No	Yes
Bernardi 2021 [23]	Cross-sectional survey	Syrian Refugees in Istanbul, Turkey.	302 Syrian refugees in Istanbul	To examine the association between COVID-19 and changes in mental health in Syrian refugees in Turkey.	July 2020 – September 2020	Yes	Yes	No	Yes
Guglielmi 2020 [24]	Mixed methods: cross-sectional survey and qualitative study	Rohingya adolescent refugees in Cox’s Bazar, Bangladesh	692 Rohingya and 1069 Bangladeshi adolescents	To explore how intersecting vulnerabilities faced by Rohingya adolescents living in Cox’s Bazar, Bangladesh, have been exacerbated during the COVID-19 pandemic.	May 2020 – June 2020	Yes	Yes	Yes	Yes
Hajjar 2021 [25]	Cross-sectional telephone survey with patients from 1 health facility	Syrian refugees in Lebanon	129 Syrian refugee families.	To assess the burden of COVID-19 by looking at the current living conditions, examining available services provided, and identifying the economic and health challenges of Syrian refugees in Lebanon.	May 2020	Yes	Yes	No	Yes
Jones 2022 [26]	Mixed methods: cross-sectional survey and qualitative study	Refugees (Syrian and Palestinian) and vulnerable Jordanian adolescents, Jordan.	3,311 surveyed total over two waves (2,574 surveyed twice)	To explore the pandemic’s effects on the psychosocial wellbeing and resilience of adolescents affected by forced displacement	October 2018 -January 2021	Yes	No	Yes	Yes
Kurt 2021 [27]	Cross-sectional survey	Syrian refugees in Turkey	345 Syrian refugees	To investigate the role of resource loss, discrimination, and social support on the psychological impacts of COVID-19 related stressors on Syrian refugees in Turkey.	September 2020 -October 2020	Yes	No	No	Yes
Lusambili 2020 [28]	Qualitative	Refugees and NGO staff in Kenya	15 patients and 10 purposively sampled staff	To improve understanding of the impact of COVID-19 on women refugees’ access to and utilisation of antenatal care, delivery and postnatal care in Eastleigh, Kenya	October 2020	Yes	Yes	Yes	Yes
Martuscelli 2020 [29]	Qualitative	Refugees in Brazil	29 refugees in Brazil	This article assesses how refugees in Brazil were affected by federal responses to the pandemic.	March 2020 - and April 2020	No	Yes	No	Yes
Moya 2021 [30]	Cohort (adapted from RCT)	IDP primary caregivers in Tumaco, Colombia	1376 primary caregivers	To analyse how the pandemic is related to early changes in mental health among caregivers	March 2018 – March 2020	Yes	No	No	Yes
Ozer 2022 [31]	Qualitative	IDPs in Burkina Faso	106 IDPs living in or adjacent to ITSs Burkina Faso	To explore how COVID-19 has affected the lives of IDPs in Burkina Faso	March 2020 - and May 2020	No	No	No	Yes
Palattiyil 2022 [32]	Mixed methods: cross-sectional survey and qualitative study	Refugees accessing HIV and TB treatment, Kampala, Uganda	229 surveyed quantitatively, 26 in-depth interview and 8 key informant interviews	To assess the impact of the Covid-19 pandemic on access to care in 4 health centres for refugee HIV and TB clinic patients in Uganda	August 2021 – October 2021	No	Yes	Yes	Yes
Palit 2022 [33]	Cross-sectional	Rohingya refugees with pre-existing health problems in Cox’s Bazar	732 in the first survey and of those 342 completing the second survey	To examine the impact of the current pandemic on the mental health of Rohingya refugees living in Bangladesh	July 2019 – November 2020	Yes	No	No	Yes
Rodo 2022 [34]	Qualitative	Humanitarian actors working in FCAS (Afghanistan, Colombia, DRC, Iraq, Nigeria, Somalia, South Sudan, Syria, Venezuela, Yemen, Zimbabwe, and Bangladesh)	39 key informant interviews (2 donor staff, 2 academics, 34 humanitarian agency staff	To investigate the collateral impact of COVID-19 on funding, services and MNCHN outcomes in FCAS, as well as adaptations used in the field to continue activities	October 2020 – February 2021	Yes	Yes	Yes	Yes
Unver 2022 [35]	Retrospective observational, single-centre cohort study	Refugee adolescents in Turkey referred for inpatient care in a psychiatric facility	236 pre-pandemic cohort 126 post-pandemics	To examine the impact on admissions to a refugee child mental health outpatient unit of the COVID-19 pandemic	March 2019 – February 2021	No	Yes	Yes	Yes
Zambrano- Barrágan 2021 [36]	Qualitative ethnographic study	Venezuelan refugees in Peru and Colombia	130 Venezuelan migrants and state and non-governmental actors	To understand how COVID-19 has affected access to healthcare among migrants in Latin American cities	July 2020 – September 2020	No	Yes	Yes	Yes

### The impact of the COVID-19 pandemic on health outcomes

Ten studies collected data on non-COVID health outcomes (Table 3). Eight related to mental health (five with adults, two with adolescents, and one related to families and children). Two studies assessed physical health outcomes in children. No studies evaluated physical health outcomes in adults.

**Table 3:** Findings on non-COVID-19 health outcomes (*N* = 10)

Author / date [ref]	Study population/ setting	Outcomes assessed (instrument used)	Results
Akhtar 2021 [22]	Syrian refugees in Jordan	Depression (HSCL-25)	No effect on depressive symptoms pre vs. post-Covid-19-pandemic
		Anxiety (HSCL-25	No effect on anxiety symptoms pre vs. post-Covid-19-pandemic
		PTSD (PCL-5)	Improvement in PTSD symptoms pre vs. post-Covid-19-pandemic: 16.01 to 5.85 post-Covid-19-pandemic (p value not reported)
Bernardi 2021 [23]	Syrian Refugees in Turkey	Depression (CESD-10)	Positive association with increased Covid-1919 disruption
		Anxiety (GAD-7)	Positive association with increased Covid-19 disruption
Guglielmi 2020 [24]	Rohingya adolescents in Bangladesh	Depression (PHQ-8)	Prevalence of 6.2% qualitatively linked to pandemic-related disruption depression during the pandemic. Contrasts with 3.7% depression prevalence among host community Bangladeshi adolescents in urban accommodation.
Hajjar 2021 [25]	Syrian refugees in Lebanon	Self-reported family stress (no instrument used)	88% reported constant stress. Common problems among children: anxiety, aggressiveness, irregular sleep, and hyperactivity.
Jones 2022 [26]	Syrian and Palestinian adolescent refugees, vulnerable Jordanians, Jordan.	Depression (PHQ-8)	Prevalence of moderate-to-severe depression amongst adolescents improved between May 2020 and January 2021 by 3.2pp (*p* < .001). When disaggregated, Syrian adolescents living among host communities had the highest prevalence of moderate to severe depression at 16% at R1 and 13% at R2 (p = < 0.05), and those in informal tented settlements had the lowest prevalence of 13% at R1 and 8% at R2(p = < 0.05). Qualitatively linked to easing of lockdown restrictions
		Anxiety (GAD-7)	Jordanian adolescents were 7.7pp (*p* = .05) more likely to report increased anxiety due to the COVID-19 pandemic and 20.9pp (*p* = .001) more likely to report thoughts of self-harm due to the pandemic than Syrian refugees in refugee camps in Jordan (34). These adolescents described the causes of their anxiety as reduced privacy and increasing tension in the home due to lockdowns, fear of catching COVID-19, and reduced social relationships.
Kurt 2021 [27]	Syrian refugees in Turkey	Depression (PHQ-9) Anxiety (GAD-7)	Prevalence 52.9% depression symptoms. Prevalence of 42.9% anxiety symptoms. Positive associations between COVID-19-induced resource loss and perceived discrimination and depressive and anxiety symptoms (β = 0.179, *p* < .01, β = 0.223, *p* < .001) (β = 0.151, *p* < .01, β = 0.211, *p* < .001) (33). Decreased social support significantly negatively predicted symptoms of depression (β = -0.233, *p* < .001) and anxiety (β = -0.231, *p* < .001). High levels of social support (1 SD above the mean) did not lead to any significant association between resource loss and depressive symptoms (β = 0.060, *p* = .405) and anxiety symptoms β = 0.031, *p* = .677
Lusambili 2020 [28]	Refugees & NGO staff in Kenya	Child health	Reported decrease in childhood immunisations due to the pandemic
Moya 2021 [30]	IDP primary caregivers, Colombia	Depression (Symptoms checklist 90-adapted) Anxiety (Symptoms checklist 90 -adapted)	Increased likelihood of reporting symptoms above the risk threshold by 5 pp (95% CI 0.4–10) for depression and 14 pp for anxiety (95% CI 10–18).
		Parenting stress (Parenting Stress Index)	Increased likelihood of reporting symptoms above the risk threshold
Palit 2022 [33]	Rohingya refugees in Bangladesh	Stress (RHS-15 parts I & II)	Surveys in July 2019 and November 2020 found a significant worsening in stress scores (Part I: 22.96 to 46.72 *p* < .001; and RHS-15 Part II: 4.43 vs. 6.91 *p* < .001.
Rodo 2022 [34]	Humanitarian actors (multiple county settings)	Child health	Reported increase in morbidity and mortality due to the pandemic

#### Adult mental health

Five studies assessed adult mental health, and the main outcomes evaluated were symptoms of depression and anxiety [22, 23, 27, 30], post-traumatic stress disorder (PTSD) [22], stress and quality of life [33]. The pandemic was associated with worse mental health symptoms in most of the studies. A cohort study by Moya et al. with IDP primary caregivers (*N* = 1376) in Tumaco, Colombia observed increased depression symptoms after the pandemic compared to prior to it (5 pp increase (95% CI 0.4–10) for depression and 14 pp increase for anxiety (95% CI 10–18)) [30]. In addition, three cross-sectional surveys (two with Syrian refugees in Turkey, and one with Rohingya refugees in Bangladesh) observed negative effects on depression, anxiety, stress and quality of life from disruptions caused by the pandemic such as resource loss, stress, and discrimination [23, 27, 33]. In contrast, a cohort study by Akhtar et al. with Syrian refugees (*N* = 410) in Azraq camp in Jordan reported improvements in PTSD (PCL-5 score change from 16.01 to 5.85 post-COVID, but *p* value not reported) and which the authors speculated was due to lockdowns reducing the potential for triggers and normalising a more restricted lifestyle which may have been reassuring for study participants [22]. The same study also found no effect on depression symptoms pre- and post-COVID-19 pandemic.

#### Adolescent mental health

Two studies assessed depression in adolescents [24, 26]. Jones et al. conducted a mixed methods study in Jordan with a group of Syrian refugees, stateless Palestinians, and vulnerable Jordanian adolescents (*N* = 3,311) [26]. The prevalence of moderate-to-severe depression amongst adolescents improved between May 2020 and January 2021. When disaggregated, Syrian adolescents living among host communities had the highest prevalence of moderate to severe depression. In qualitative interviews, those with symptoms of depression cited resource loss as one possible cause [26]. Guglielmi et al. conducted a mixed methods assessment with Rohingya adolescents (*N* = 692) assessing factors that impacted their well-being in Cox’s Bazaar, Bangladesh [24]. They reported that the adolescents were more likely to exhibit moderate-to-severe signs of depression during the pandemic (6.2%) than host community Bangladeshi adolescents in typical urban accommodation (3.7%), using the same outcome measure as Jones et al. (the PHQ-8) [24]. 

#### Family and children

A study by Hajjar et al. of Syrian refugees (*N* = 129) in Lebanon focused on family well-being and children in families [25]. It noted that 88% of respondents reported constant stress, and common problems among the children included anxiety, aggressiveness, irregular sleep, and hyperactivity. However, no outcome instruments were used, and it was from a convenience sample of health care users at a single health care facility, and so is subject to major limitations.

#### Physical health

Two studies evaluated child physical health outcomes. No studies addressed adult physical health. Rodo et al. conducted qualitative interviews with 39 humanitarian actors working in fragile and conflict-affected states (FCAS) engaged in Maternal, Newborn and Child Health and Nutrition (MNCHN) programming during COVID-19 [34]. They noted that respondents in their FCAS reported increased child morbidity and mortality due to COVID-19, with a commonly cited cause being late presentation at health facilities by more children with advanced illnesses and more severe malnutrition due to COVID-19 and related restrictions [34]. A study by Lusambili et al. with 25 staff and refugee patients in Kenya accessing maternal, child and nutrition services also observed late presentation as a critical issue, including the effects of suspension of outreach programs, reduction in child health facilities, and disruption of Vitamin A injection schedules [28]. Both studies noted the effects of interrupted vaccine campaigns, with Rodo et al. reporting outbreaks of measles and rising cases of diphtheria, tetanus, pertussis, and vaccine-derived polio among children [34]. 

### The effect of the COVID-19 pandemic on access to healthcare

Nine studies reported on access to healthcare, all finding evidence of increased barriers to accessing healthcare due to COVID-19 (Table 4) [23–25, 28, 29, 32, 34–36]. 

**Table 4:** Findings on impact on access to care (*N* = 9)

Author, year, [ref]	Study population/ setting	Entitlement	Affordability	Availability	Accommodation	Acceptability	Accessibility
Bernardi 2021 [23]	Syrian Refugees in Istanbul	Yes	No	No	No	Yes	No
Guglielmi 2020 [24]	Rohingya adolescents in Bangladesh	No	No	No	No	No	No
Hajjar 2021 [25]	Syrian refugees in Lebanon	No	Yes	No	No	No	No
Lusambili 2020 [28]	Refugees & NGO staff in Kenya	No	No	Yes	No	No	No
Martuscelli 2020 [29]	Refugees in Brazil	Yes	Yes	No	No	Yes	Yes
Palattiyil 2022 [32]	Refugees in Uganda	No	Yes	Yes	Yes	Yes	Yes
Rodo 2022 [34]	Humanitarian actors (multiple county settings)	No	No	Yes	Yes	Yes	No
Unver 2022 [35]	Refugees in Turkey	No	No	Yes	Yes	No	Yes
Zambrano-Barrágan 2021 [36]	Refugees in Peru and Colombia	Yes	Yes	No	Yes	Yes	Yes

#### Entitlement

Three studies commented on individuals’ legal status concerning COVID-19 and healthcare [23, 29, 36]. A qualitative study by Zambrano-Barrágan of Venezuelan refugees in Peru described healthcare entitlement extending to cover everyone, irrespective of migration or asylum status, during COVID-19 [36]. However, significant barriers remained, with healthcare staff frequently either unaware of this legal entitlement or discriminating against Venezuelans and thus preventing access. The study by Bernardi et al. with Syrian refugees in Istanbul found significant concerns about legal status were commonly cited by refugees as preventing care-seeking [23]. A qualitative study by Martuscelli study with refugees in Brazil found refugees were uncertain about their eligibility and access to benefits, including health care [29]. 

#### Affordability

Four studies reported how the COVID-19 pandemic-related poverty made care unaffordable for patients [25, 29, 32, 36]. Palattiyil’s mixed-methods study with refugees in Uganda reported that even if care for some health conditions was free, medications for unsubsidised conditions became unaffordable [32]. The study by Zambrano-Barrágan et al. with Venezuelan refugees in Peru and Colombia describes the complexities of navigating health care and resulting harms [36]. More than half of the 19 Venezuelans interviewed in Peru reported self-medicating at pharmacies instead of seeing doctors for HIV, TB, diabetes and other conditions. Martuscelli found that the COVID-19 pandemic affected access to the emergency benefits created by the Brazilian government to support vulnerable people [29]. The study by Hajjar of Syrian refugees in Lebanon noted that 54% of families could not afford medication, physiotherapy or medical equipment, and 43% could only partially afford these services [25]. 

#### Availability

Four studies described increased difficulties in accessing care during the pandemic period [28, 32, 34, 35]. Closures and reduced care affecting TB and HIV care, vaccination campaigns, outpatient and inpatient child health services, psychiatric services, and entire health centres were described in the studies, including interrupted essential medicine supplies [28, 32, 34, 35]. 

#### Accommodation

Four studies found evidence of service adaptations to maintain access [32, 34–36]. Examples were given of decentralising services and prioritising self-care, such as mothers performing mid-upper arm circumference measures to identify malnutrition [34]. Two studies described remote prescribing, longer prescriptions, and medication delivery [32, 35]. Technological adaptations, including telemedicine and WhatsApp communications, were used in some instances to maintain services and overcome information barriers [34, 36]. 

#### Acceptability

Five studies addressed acceptability [23, 29, 32, 34, 36]. Four studies reported that discrimination from staff and poor manner of treatment when attending healthcare directly negatively impacted their access and treatment [23, 29, 32, 36]. Bernardi et al. reported increased experience by Syrian refugees of discrimination from health care workers in Turkey during the COVID-19 pandemic [23]. In Peru, over half of participants in Zambrano-Barragán’s ethnographic study stated they had been discriminated against when accessing care, but it was unclear to what degree COVID-19 had influenced this [36]. In Brazil, there was concern by refugees that health care workers would discriminate against them when accessing treatment for COVID-19 [29]. In Uganda, adaptations such as social distancing also acted as a barrier to care for refugees. They also reported concern about discrimination in health care but it was not clear how COVID-19 may have influenced this [32]. 

#### Accessibility

Four studies described accessibility as a barrier to accessing care [29, 32, 35, 36]. In Turkey and Uganda, studies described reduced transport and long distances to facilities preventing access to care for refugee patients [32, 35]. Palattiyil et al. observed a loss to follow-up for HIV and TB refugee patients in Uganda when outreach health care teams were stopped [32]. Martuscelli found evidence of increased difficulty by refugees in Brazil accessing care due to lockdowns and fears that xenophobia and discrimination when accessing healthcare services would further increase during the COVID-19 pandemic [29]. 

### The effect of the COVID-19 pandemic on health systems

The impacts of the COVID-19 pandemic on health systems are summarised in Table 5. Seven studies reported findings relating to the impact of COVID-19 on health systems [24, 26, 28, 32, 34–36].

**Table 5:** Findings on impacts on health systems (*N* = 7)

Author, year [ref]	Study population/ setting	Medical products	Health workforce	Information	Service delivery	Leadership/ governance	Financing
Guglielmi 2020 [24]	Rohingya adolescents in Bangladesh	No	No	Yes	Yes	Yes	No
Jones 2022 [26]	Syrian and Palestinian refugees, vulnerable Jordanians, Jordan.	No	No	No	Yes	No	No
Lusambili 2020 [28]	Refugees & NGO staff in Kenya	Yes	No	No	Yes	No	No
Palattiyil 2022 [32]	Refugees in Uganda	No	Yes	Yes	Yes	No	No
Rodo 2022 [34]	Humanitarian actors (multiple county settings)	Yes	Yes	Yes	Yes	Yes	Yes
Unver 2022 [35]	Refugees in Turkey	Yes	No	Yes	Yes	Yes	No
Zambrano-Barragán 2021 [36]	Venezuelan refugees in Peru and Colombia	Yes	No	No	Yes	No	No

#### Medical products, vaccines & Technology

Supply issues of protective personal equipment, vaccines and technology were described in three studies with refugees in Kenya, Turkey, Peru and Colombia [28, 35, 36], and in one study by humanitarian actors relation to multiple FCAS [34]. 

### Health Workforce

Two studies described the health of the healthcare workforce as a factor in patients’ access to healthcare [32, 34]. Staff falling ill with COVID-19 and transport disruption impacted services for refugees in Uganda [32]. Furthermore, organisations reallocated staff to COVID-19-related duties to the detriment of child health and nutrition services in multiple FCAS [34]. 

#### Information

Four studies identified conflicting communications about COVID-19 and whether to attend health services [24, 32, 34, 35]. One example was an NGO in South Sudan attributing a drop in service use to mixed messaging, as organisations told people to isolate but also attend normal appointments [34]. The same study gave an example from a health worker in Yemen on how community engagement successfully combated mixed messaging and maintained healthcare attendance [34]. The study by Guglielmi with Rohingya adolescents in Cox’s Bazar in Bangladesh found that COVID-19 messaging increased Rohingya refugees’ fears of attending health services for non-COVID-19 treatment [24]. The study Unver et al. with Turkish adolescent psychiatric service users noted how service users felt that the Turkish government’s advice to cancel health appointments contributed to reduced psychiatric service use [35]. 

#### Service Delivery

Seven studies examined the impact of COVID-19 on service delivery [24, 26, 28, 32, 34–36]. The study by Rodo et al. with NGO staff noted altered or absent services due to COVID-19 in multiple FCAS, including interrupted vaccine services for polio and measles, and suspension of new-born care in Bangladesh and Somalia [34]. Other studies reported attrition in TB and HIV services for refugees in Uganda [32]. Venezuelan refugee also reported that the quality of health services had worsened during the pandemic [36]. 

#### Leadership & Governance

The lack of a strategy addressing COVID-19 while maintaining service continuity was noted by respondents in multiple FCAS in the study by Rodo et al. [34] The impact of mixed messaging between encouraging to isolate but also seek health care was highlighted in studies with Rohingya refugees in Bangladesh and Syrian refugees in Turkey, including conflicting messaging between NGOs and stage actors [24, 35]. 

#### Financing

The study in multiple FCAS commented on structural funding issues due to the COVID-19 pandemic at a health systems level, most notably diversion of funds from critical health care and public health activities to COVID-19 related activities [34]. It was also noted that once emergency funding became available, it was often earmarked for COVID-19-specific activities and did not replace the lost funding for standard health activities.

### Influence of the COVID-19 pandemic on the social determinants of health

All fifteen studies reported the impacts of the COVID-19 pandemic on the social determinants of health [22–36]. (Table 6).

**Table 6:** Findings on social determinants of health (*N* = 15)

Study Author [ref]	Study Population and setting	Gender	Ethnicity	Age	Poverty	Food insecurity	Housing	Education
Akhtar 2021 [22]	Syrian refugees in Jordan	Yes	No	No	No	No	No	No
Bernardi 2021 [23]	Syrian refugees in Istanbul	Yes	Yes	No	No	No	No	No
Guglielmi 2020 [24]	Rohingya refugees in Bangladesh	Yes	No	No	Yes	Yes	No	Yes
Hajjar 2021 [25]	Syrian refugees in Jordan	No	No	No	Yes	Yes	No	Yes
Jones 2022 [26]	Syrian & Palestinian refugees; vulnerable Jordanians, Jordan	Yes	No	No	Yes	Yes	Yes	Yes
Kurt 2021 [27]	Syrian refugees in Turkey	Yes	Yes	Yes	Yes	No	No	Yes
Lusambili 2020 [28]	Refugees & NGO staff, Kenya	Yes	No	No	Yes	Yes	No	No
Martuscelli 2020 [29]	Refugees in Brazil	No	Yes	No	Yes	Yes	No	Yes
Moya 2021 [30]	Primary caregivers in Colombia	Yes	No	No	Yes	Yes	No	No
Ozer 2020 [31]	IDPs in Burkina Faso	No	No	No	Yes	Yes	No	Yes
Palattiyil 2022 [32]	Refugees in Uganda	Yes	Yes	No	Yes	Yes	No	No
Palit 2022 [33]	Rohingya refugees in Bangladesh	Yes	No	Yes	No	No	No	No
Rodo 2022 [34]	Humanitarian actors working in various FCAS	Yes	No	No	No	Yes	No	No
Unver 2022 [35]	Refugees in Turkey	Yes	No	No	No	No	No	No
Zambrano- Barragán 2021 [36]	Venezuelan refugees in Peru and Colombia	Yes	Yes	No	Yes	Yes	Yes	No

#### Gender

Twelve studies noted gender differences in the impacts of the pandemic, with mixed outcomes [22–24, 26–28, 30, 32–36]. Three studies found no association between gender and anxiety or depressive symptoms [22, 23, 27]. However, in the study by Palit et al., Rohingya refugee women in Bangladesh had significantly higher stress scores than men (*N* = 732) [33]. Lockdown restrictions had distinct impacts on girls, for example for refugees in Jordan who cited menstrual hygiene taboos as increasing their stress now that homes were more crowded [26]. One study found that respondents felt domestic violence had increased in refugee households in Bangladesh during the pandemic [24]. Venezuelan refugee married girls (females below the age of 18) were also significantly less likely to be able to access healthcare [36]. One study found Syrian refugee boys in Jordan were more likely to be victims of violence in the household (52.5% vs. 46.7% of girls, *p* < .001) [26]. Guglielmi et al. noted concern that the programs for refugee women’s empowerment and to support victims of gender-based violence were closed in Cox’s Bazar in Bangladesh, which may have compounded their disadvantages [24]. Rodo et al. highlighted the interruption of gender-based violence prevention programs due to the pandemic in several FCAS [34]. However, Unver et al. noted that, before the COVID-19 pandemic, referrals to their refugee mental health clinic were predominantly female but equalised by gender during the pandemic [35]. 

#### Ethnicity

Discrimination based on ethnicity was found to be increased during the pandemic in five studies [23, 27, 29, 32, 36]. The study by Palit et al. of refugees in Uganda noted in qualitative interviews that people felt that refugees were spreading COVID-19 and that locals believed that refugees had more money than locals which was a source of animosity and discrimination [32]. Martuscelli found evidence of discrimination against Venezuelan refugees specifically preventing them accessing care in Brazil [29]. 

#### Age

Older age was positively associated with increased social support among Syrian refugees in Turkey, which was a protective factor against mental illness [27]. Younger age correlated with increased stress levels before and after the COVID-19 pandemic among Rohingya refugees in Bangladesh [33]. 

#### Poverty

Poverty worsened during the pandemic in ten studies [24–32, 36]. Eight studies attributed this to pandemic-related job loss [24–28, 30, 32, 36], such as the ‘hawkers’ described in the Ugandan study by Palattiyil et al., as lockdown restrictions prevented them from working [32]. In the qualitative study with Venezuelan refugees, participants reported losing 50–80% of their daily income due to the pandemic [36]. The study by Ozer et al. of IDPs in Burkina Faso reported how the lockdown there had resulted in a significant decrease in income-generating activities for IDPs, leading to economic hardships and increased poverty, with 85% of respondents having no income-generating activities at that point in the pandemic. It also reported that authorities and humanitarian actors had to reduce their humanitarian assistance to the IDPs due to the pandemic, and so further increasing poverty levels [31]. The study by Hajjar et al. of Syrian refugees in Lebanon reported that 79% of respondents had lost their jobs during the pandemic. Of those who had kept their jobs, 68% had their wages reduced [25]. The study reports that 45% of participants could not afford basic needs (food, shelter, clothes) during the crisis. Additionally, 92% of families had new financial debts during COVID-19.

#### Food Insecurity

Food insecurity rose due to the COVID-19 pandemic in ten studies [24–26, 28–32, 34, 36], attributed mainly to rising poverty. The study with Venezuelan refugees in Peru found 74% of Venezuelans could not afford food and necessities two months into the start of the pandemic [36]. IDPs in Burkina Faso reported decreases in the quantity, quality, and frequency of food assistance received, resulting in food insecurity [31]. The study by Hajjar et al. of Syrian refugees in Lebanon reported that 55% of families could only partially afford basic needs, including food [25]. 

#### Housing

The study of Venezuelan refugees in Peru and Colombia reported rising evictions, with 37% of migrants in Peru being unable to pay rent [36]. A study in Jordan showed the influence of housing, with levels of depression, anxiety and thoughts of self-harm lower for adolescent refugees in Syrian refugee camps compared to Syrian refugees in urban settings [26]. The authors suggested there may be protective social benefits compared with refugees in urban environments who may have faced more isolation and barriers to support during the pandemic [26]. 

#### Education

Six studies addressed education as a health determinant [24–27, 29, 31]. All reported an impact on education. For example, the study by Hajjar et al. of Syrian refugees in Lebanon reported that 70% of children did not continue their education at home during the pandemic, with the main reason being lack of access to online resources (65%) and devices (laptops, smartphones) (35%) [25]. 

There were several potential social determinants that were not addressed in the eligible studies, such as social exclusion, structural violence, and expenditure on health. Other social determinants, such as discrimination, were raised in studies in relation to acceptability and access to health services and so covered in those sections above.

### The quality of the evidence

Of the quantitative studies, only three were rated as good quality [22, 26, 30]. Common weaknesses in the other quantitative studies included biases in the sample selection (including reliance on convenience sampling), poor sampling explanation, unjustified sample sizes, and limited generalisability (e.g. based on a sample from a single health facility). Explanations for the non-response rate for those invited to participate in surveys were missing in several studies. Some of the limitations in the qualitative studies included insufficient information on recruitment process, lack of reflection on the relationship and power dynamics between the researchers and participants. Further details on the quality appraisal of individual studies are provided in Supplementary Material 3.

## Discussion

To the best of our knowledge, this is the first systematic review to assess the indirect health impact of the COVID-19 pandemic on crisis-affected populations in LMICs (excluding our related SRH review [13]). Fifteen studies met our eligibility criteria. Ten studies assessed non-COVID-19 health outcomes – eight related to mental health and two to physical health. The studies on mental health generally suggested negative impacts of the pandemic on mental health outcomes. A global survey of migrants also found negative mental health impacts on migrants and refugees due to the pandemic [37]. These negative impacts risk compounding already elevated mental health needs among crisis-affected populations [38]. Critically, no studies evaluated adult physical health outcomes in crisis-affected populations. The absence of studies on NCD outcomes is particularly surprising given the additional risks of COVID-19 for people living with NCDs and rising concern about NCDs in crisis-affected settings [39, 40]. This perhaps reflects the limited investment towards NCDs in crisis-affected settings and the need to adapt services and models of care for such settings [41–43]. The only physical outcomes researched were related to child nutrition and new-born care. The two papers examining this suggested worsening trends in child nutrition levels, vaccination rates and general morbidity and mortality [28, 34]. Other studies have observed worsening malnutrition during the pandemic due to food chain disruption and interrupted nutrition activities [44–46]. The findings in this review regarding vaccine-preventable disease outbreaks due to immunisation program disruptions are also supported by emerging evidence from academics and humanitarian actors [47, 48]. 

Studies identified in our review documented the negative impact of the pandemic on accessing and delivering health services. We identified consistently negative impacts across criteria of eligibility, accessibility, affordability, availability, and acceptability. This reflects findings from crisis-affected settings observed in studies since our review was conducted [49–51]. Studies from high-income countries and LMICs have recorded excess morbidity and mortality during the pandemic (from non-COVID outcomes) due to restricted access to health care [52], but the evidence identified in our review did not measure excess morbidity and mortality. As a result, comparisons cannot made with these studies from LMICs or high-income countries. It is recommended that new methods for determining excess mortality from crises such as COVID-19 in crisis-affected settings be applied in future disease outbreaks [53, 54]. The identified studies in our review did note service adaptations to support access, such as self-care and family care, remote and longer-term prescribing, home delivery for medications, and increased use of technology such as for telemedicine. These potentially positive adaptations and use of technology could be further developed to help increase access to health care for crisis-affected populations, but evaluations are required to better understand the implementation, acceptability, effectiveness, and equity implications of these adaptations [55]. 

COVID-19 harmed health system elements. We reported conflicting messages and information about isolation and seeking health care, disruption to the healthcare workforce, failures in logistics and supplies, and extensive disruption of services due to the pandemic. These findings reflect those from global studies on health service and system challenges during the COVID-19 pandemic which identified health workforce challenges, lack of funding, and shortages of supplies and equipment as critical bottlenecks in care provision [56]. The observation that staff in several NGOs reported limited strategic oversite and mixed messaging reflects findings from global reviews on conflicting leadership strategies and funding priorities in LMICs during the pandemic [57]. Evidence was also not identified on health system resilience and there is a need for more research on this in crisis-affected settings to support more effective health system responses, leadership and decision-making [58, 59]. Studies with crisis-affected populations, such as in Eastern Democratic Republic of Congo, have highlighted how violence, mobility restrictions, and resource availability impede access to services. They also provide key recommendations for adapting services and system capacity to mitigate the effects of insecurity, and these recommendations could be helpful in crisis-affected settings also affected by pandemics [60, 61]. 

Almost all the studies in our review found some suggestion that health inequalities widened due to COVID-19 based on determinants such as gender, ethnicity, poverty, food insecurity, and education. This evidence was limited in scope and depth, but substantial research from high- and middle-income settings has highlighted the role of inequalities in health outcomes during the pandemic [62, 63]. This finding reinforces the need to address equity in humanitarian and pandemic responses, including for supporting health systems resilience and strengthening [64, 65]. 

The quantity and quality of evidence were limited, but the challenges of conducting research during the pandemic should be acknowledged. Overall, the limited evidence base supports calls for improved guidance on the collection and use of evidence in pandemics that is accessible, coherent, and contextually relevant [66, 67], and the need for a cohesive strategy to identify and action research priorities in crisis-affected settings during future pandemics.

We highlight four key recommendations stemming from our findings. First, to evaluate ongoing adaptations to service delivery initially made during COVID-19, such as self-care and family care, remote and longer-term prescribing, home delivery for medications, and increased use of technology such as for telemedicine. Second, to recognise and plan for health system impacts from pandemics in crisis-affected areas, learning from adaptations made to health systems in crisis-settings. This includes having strategic plans to support health system resilience and more effective responses, financing, staff support, leadership, and decision-making. Third, recognise and address the risk of pandemics further widening inequalities among crisis-affected populations and ensure that response planning addresses the social determinants of health. Fourth, the limited evidence identified highlights the need for the stronger collection and use of data with crisis-affected populations during pandemics, including having a strategy for prioritising research and data collection.

### Limitations

There are several limitations to this review. To capture a comprehensive range of studies, we did not impose a quality threshold on studies and including studies rated poor quality could be a limitation. Given the exploratory nature of the review and diverse range of methods and health topics, a meta-analysis was not possible. The small number of eligible studies also limits the generalisability of the review findings. Our search end date was 31 July 2022 and there will have been studies published since then (for example [49–51]). Another potential limitation is the use of the Penchansky and Thomas framework rather than more recent frameworks that focus more on integrating health service demand and supply-side-factors [68]. Finally, our search was limited to English-language studies only.

## Conclusion

There was limited evidence identified on the indirect health impacts of the COVID-19 pandemic on crisis-affected people in LMICs, particularly in relation to physical health effects. More consistent evidence was identified on barriers to accessing care, damage to health systems and social determinants of health. Of note is the need for greater health leadership, staffing support, and funding continuity in future pandemics. A cohesive strategy should also be developed to identify and action research priorities in crisis-affected settings in future pandemics, including the use of new methods for estimating excess mortality and morbidity.

### Electronic supplementary material

Below is the link to the electronic supplementary material.

Supplementary Material 1

## Data Availability

No datasets were generated or analysed during the current study.

## Abbreviations

CASP: Critical Appraisal Skills Program
CESD-10: Center for Epidemiologic Studies Depression Scale
DRC: Democratic Republic of the Congo
FCAS: Fragile and Conflict-affected States
GAD-7: Generalised Anxiety Disorder-7
HIV: Human Immunodeficiency Virus
HSCL-25: Hopkins Symptoms Checklist-25
IDPs: Internally Displaced Persons
MNCHN: Maternal, New-born and Child Health and Nutrition
NCDs: Non-communicable diseases
NGO: Non-governmental Organisation
NOS: Newcastle Ottowa Scale
PHQ-8: Patient Health Questionnaire-8
PTSD: Post-Traumatic Stress Disorder
PRISMA: Preferred Reporting Items for Systematic Reviews and Meta-Analyses
RCT: Randomized Control Trial
RHS: Refugee Health Screening
TB: Tuberculosis
