# Supplementary material for: The effect of COVID-19 on the non-COVID health outcomes of crisis-affected peoples: a systematic review
Source: Confl Health. 2024 Apr 25;18:37. doi: 10.1186/s13031-024-00592-7 (PMC11044391; doi:10.1186/s13031-024-00592-7)
Supplement: Supplementary file 1 — Supplementary Material 1 [file 13031_2024_592_MOESM1_ESM.docx]

**Supplementary Material 1: PRISMA checklist**

| **Section and Topic** | **Item #** | **Checklist item** | **Location where item is reported** |
| --- | --- | --- | --- |
| **TITLE** | | |  |
| Title | 1 | Identify the report as a systematic review. | 1 |
| **ABSTRACT** | | |  |
| Abstract | 2 | See the PRISMA 2020 for Abstracts checklist. | 2 |
| **INTRODUCTION** | | |  |
| Rationale | 3 | Describe the rationale for the review in the context of existing knowledge. | 3 |
| Objectives | 4 | Provide an explicit statement of the objective(s) or question(s) the review addresses. | 3-4 |
| **METHODS** | | |  |
| Eligibility criteria | 5 | Specify the inclusion and exclusion criteria for the review and how studies were grouped for the syntheses. | 4 |
| Information sources | 6 | Specify all databases, registers, websites, organisations, reference lists and other sources searched or consulted to identify studies. Specify the date when each source was last searched or consulted. | 4 |
| Search strategy | 7 | Present the full search strategies for all databases, registers and websites, including any filters and limits used. | 4 |
| Selection process | 8 | Specify the methods used to decide whether a study met the inclusion criteria of the review, including how many reviewers screened each record and each report retrieved, whether they worked independently, and if applicable, details of automation tools used in the process. | 4 |
| Data collection process | 9 | Specify the methods used to collect data from reports, including how many reviewers collected data from each report, whether they worked independently, any processes for obtaining or confirming data from study investigators, and if applicable, details of automation tools used in the process. | 4 |
| Data items | 10a | List and define all outcomes for which data were sought. Specify whether all results that were compatible with each outcome domain in each study were sought (e.g. for all measures, time points, analyses), and if not, the methods used to decide which results to collect. | 4 |
|  | 10b | List and define all other variables for which data were sought (e.g. participant and intervention characteristics, funding sources). Describe any assumptions made about any missing or unclear information. | 4 |
| Study risk of bias assessment | 11 | Specify the methods used to assess risk of bias in the included studies, including details of the tool(s) used, how many reviewers assessed each study and whether they worked independently, and if applicable, details of automation tools used in the process. | 4 |
| Effect measures | 12 | Specify for each outcome the effect measure(s) (e.g. risk ratio, mean difference) used in the synthesis or presentation of results. | 4-5 |
| Synthesis methods | 13a | Describe the processes used to decide which studies were eligible for each synthesis (e.g. tabulating the study intervention characteristics and comparing against the planned groups for each synthesis (item #5)). | 4 |
|  | 13b | Describe any methods required to prepare the data for presentation or synthesis, such as handling of missing summary statistics, or data conversions. | N/A |
|  | 13c | Describe any methods used to tabulate or visually display results of individual studies and syntheses. | N/A |
|  | 13d | Describe any methods used to synthesize results and provide a rationale for the choice(s). If meta-analysis was performed, describe the model(s), method(s) to identify the presence and extent of statistical heterogeneity, and software package(s) used. | 4 |
|  | 13e | Describe any methods used to explore possible causes of heterogeneity among study results (e.g. subgroup analysis, meta-regression). | N/A |
|  | 13f | Describe any sensitivity analyses conducted to assess robustness of the synthesized results. | 4 |
| Reporting bias assessment | 14 | Describe any methods used to assess risk of bias due to missing results in a synthesis (arising from reporting biases). | N/A |
| Certainty assessment | 15 | Describe any methods used to assess certainty (or confidence) in the body of evidence for an outcome. | 4 |
| **RESULTS** | | |  |
| Study selection | 16a | Describe the results of the search and selection process, from the number of records identified in the search to the number of studies included in the review, ideally using a flow diagram. | 5 |
|  | 16b | Cite studies that might appear to meet the inclusion criteria, but which were excluded, and explain why they were excluded. | 5 |
| Study characteristics | 17 | Cite each included study and present its characteristics. | 5 |
| Risk of bias in studies | 18 | Present assessments of risk of bias for each included study. | Supplementary Material 3 |
| Results of individual studies | 19 | For all outcomes, present, for each study: (a) summary statistics for each group (where appropriate) and (b) an effect estimate and its precision (e.g. confidence/credible interval), ideally using structured tables or plots. | NA |
| Results of syntheses | 20a | For each synthesis, briefly summarise the characteristics and risk of bias among contributing studies. | Supplementary Material 3 |
|  | 20b | Present results of all statistical syntheses conducted. If meta-analysis was done, present for each the summary estimate and its precision (e.g. confidence/credible interval) and measures of statistical heterogeneity. If comparing groups, describe the direction of the effect. | N/A |
|  | 20c | Present results of all investigations of possible causes of heterogeneity among study results. | N/A |
|  | 20d | Present results of all sensitivity analyses conducted to assess the robustness of the synthesized results. | N/A |
| Reporting biases | 21 | Present assessments of risk of bias due to missing results (arising from reporting biases) for each synthesis assessed. | N/A |
| Certainty of evidence | 22 | Present assessments of certainty (or confidence) in the body of evidence for each outcome assessed. | 12-13 |
| **DISCUSSION** | | |  |
| Discussion | 23a | Provide a general interpretation of the results in the context of other evidence. | 13-15 |
|  | 23b | Discuss any limitations of the evidence included in the review. | 15 |
|  | 23c | Discuss any limitations of the review processes used. | 15 |
|  | 23d | Discuss implications of the results for practice, policy, and future research. | 15 |
| **OTHER INFORMATION** | | |  |
| Registration and protocol | 24a | Provide registration information for the review, including register name and registration number, or state that the review was not registered. | N/A |
|  | 24b | Indicate where the review protocol can be accessed, or state that a protocol was not prepared. | N/A |
|  | 24c | Describe and explain any amendments to information provided at registration or in the protocol. | N/A |
| Support | 25 | Describe sources of financial or non-financial support for the review, and the role of the funders or sponsors in the review. | 17 |
| Competing interests | 26 | Declare any competing interests of review authors. | 16 |
| Availability of data, code and other materials | 27 | Report which of the following are publicly available and where they can be found: template data collection forms; data extracted from included studies; data used for all analyses; analytic code; any other materials used in the review. | 16 |

**Supplementary Material 2: Search terms**

| Field One: Terms relating to crisis-affected peoples | Field Two: Terms relating to COVID-19 |
| --- | --- |
| (Refuge* or evacue* or asylum seeker* or evacuated or internally displaced perso* or internally displaced peopl* or IDP or forced migrant* or crisis affected or humanitarian or humanitarian cris* or humanitarian relief or foreign aid or humanitarian or humanitarian agenc* or Disaster* relief or Disaster* plan or Relief Work or Rescue Work or emergency response or war or conflict or armed zone or conflict zone or Disaster Medicine or mass casualty incident* or responder* or starvation or famine or aid work or natural disaster*) | (COVID 19 or SARS-CoV-2 or coronavirus or 2019-nCoV or corona virus or pandemic) |
| Field One combined with Field Two using ‘And’ | |

**Supplementary Material 3: Critical Appraisal of Studies**

Newcastle Ottowa Scale (NOS) for cross-sectional and cohort studies

| Study | Selection | Comparability | Outcome | Total | Score | Notes |
| --- | --- | --- | --- | --- | --- | --- |
| Cross-Sectional: | Max = 5 | Max = 2 | Max =3 | Max = 10 |  |  |
| Bernardi | 2 | 2 | 2 | 6 | Fair | 70% response on second survey, face-to-face interviews CRISIS, CESD10, STAI and PS-4 tools used. Sample size from Frekhan's randomised cohort of Syrian refugees living in turkey. 302 responses not justified from sample of 1200 invited. |
| Guglielmi | 1 | 1 | 1 | 3 | Poor | Gage data + 7 KII + 30 IDI mixed methods: No baseline data for cohorts by demographics - not possible to assess for generalisability or bias. no attempt to do regression analysis or variables controlled for with p values for all results, mixed outcomes, no predefined rationale for not presenting nonsignificant findings not clear. |
| Hajjar | 0 | 1 | 1 | 2 | Poor | Limitations: convenience sampling of patients at a single facility, sample size justification, non-comparability between respondents and non-respondents, outcome measurement, and the reporting of confidence intervals and p-values. |
| Jones* | 3 | 2 | 3 | 8 | Good | Good |
| Kurt | 1 | 2 | 1 | 4 | Poor | Financial incentive used to recruit people via Facebook with 6$ grocery token - highly biased sampling method with no verification of refugee status in the context of offering an incentive online. |
| Palattiyil* | 1 | 1 | 2 | 3 | Poor | Purposive sampling no recording of those who declined. 78% female participants not adequately controlled for. |
| Palit | 1 | 2 | 2 | 5 | Poor | Excluded pre-existing MH, pregnant and lactating women - unjustified. At health centre so not representative sample. Statistically significant different between RHS 15 in the pre and post pandemic cohort (over 50% loss to follow-up, characteristics unconvincingly accounted for) |
| Cohort: | Max=4 | Max=2 | Max=3 | Max=9 | Score |  |
| Akhtar | 4 | 2 | 3 | 9 | Good | RCT NB originally an RCT adapted to cohort difficult to assess in those parameters. Technically comparable cohort but limited generalisability |
| Moya | 4 | 2 | 2 | 8 | Good | RCT More beneficiaries of cash transfers (sig) in post pandemic group (mixed effect) 2-5 weeks v short timeline to results. More IDPs pre pandemic. NB originally an RCT adapted to cohort difficult to assess in those parameters. Technically comparable cohort but limited generalisability |
| Unver | 2 | 2 | 2 | 6 | Fair | samples size small 236 - 125 pre post pandemic. Must have gov ID card for referral so not representative sample for refugees, decreased dep and anxiety referrals. Poor comparability, limited utility given retrospective observational single-centre design. |

Critical Appraisal Skills Program (CASP) for qualitative studies

|  | **Question 1** | | **Question 2** | | **Question 3** | | **Question 4** | | **Question 5** | | **Question 6** | | **Question 7** | | | **Question 8** | | **Question 9** | | **Question 10** | |
| --- | --- | --- | --- | --- | --- | --- | --- | --- | --- | --- | --- | --- | --- | --- | --- | --- | --- | --- | --- | --- | --- |
| Author | Clear research aims? | | Is method appropriate? | | Research design appropriate to  address research aims? | | Recruitment strategy appropriate to research aims? | | Data collected in a way that addressed  the topic? | | Relationship between researcher/ participant considered enough? | | Ethical issues been taken into consideration? | | | Data analysis sufficiently rigorous? | | Clear statement of findings? | | Value of research? | |
|  | Y/N | Note | Y/N | Note | Y/N | Note | Y/N | Note | Y/N | Note | Y/N | Note | Y/N | Note | | Y/N | Note | Y/N | Note | Y/N | Note |
| Guglielmi | Y |  | Y |  | Y |  | N | Initial sample was random from cohort but unclear | ? | Tool for interviews not given | N | No description of controlling for dynamics. | N | Translation issues re Rohingya dialect and interpreters | | Y | Not cross-checked | N | Unclear reporting of findings | N | Lacks detail in collection and analysis. |
| Jones | Y |  | Y |  | Y |  | Y |  | Y |  | ? | Sampling and implications unclear. | Y |  | | Y | Clear rigorous standards applied | Y | Detailed findings and interpretation |  | Very valuable locally, Some generalisability due to large sample size and strong thematic analysis |
| Lusambili | Y |  | Y |  | Y | Good spread of CHW vs patients from pre and post | Y | methods explicit | Y | Direct to needs of research question | N | Noted issues with interpretation limitations with data from mothers. | Y |  | | Y | Detailed description of NVivo use coding process | Y |  | Y | Valuable and robust. |
| Martuscelli | Y |  | Y |  | Y |  | N | Insufficient information on recruitment | Y |  | N |  | N | Insufficient information on ethics | | N | Insufficient information on analysis | Y |  | Y | Useful findings but limited by methodological limitations. |
| Ozer | Y |  | Y |  | Y |  | Y |  | Y |  | Y |  | Y |  | | N | Insufficient information on analysis | Y |  | Y |  |
| Palattiyil | Y |  | Y |  | Y |  | ? | Insufficient information | Y |  | ? | No comment provided | Y |  | | N | No substantial comment from Authors | N |  | N | Appropriate design but qual. research lacks detail in collection and analysis. Gaps in the logic of how qualitative data was presented. |
| Rodo | Y |  | Y |  | Y | detailed assessment | Y | purposive sampling used | Y | Direct to needs of research question | Y | Acknowledged power between HIC researchers and research in LMIC | Y |  | | Y | Software/ framework Coding cross-checking | Y | Y | Y | Good quality methods. Detailed results. |
| Zambrano | Y |  | Y |  | Y |  | Y |  | Y |  | Y |  | Y | |  | Y |  | Y |  | Y | Only interviews only with NGO staff. Large range of countries and settings so somewhat generalisable. |
